# From the lens of early-career researchers: bridging science, technology, arts, and humanities to tackle antimicrobial resistance

**DOI:** 10.1038/s41467-025-67863-6

**Published:** 2026-01-03

**Authors:** Nikhil Bhalla, Mojgan Rabiey, Prachi Bendale, Katie Lawther, Janice Spencer, Alberto Longo, Lucky Lucky, Vishal Chaudhary, Paul McCormack, Saikat Jana, Patrick S. M. Dunlop, Linda Oyama

**Affiliations:** 1 Nanotechnology and Integrated Bioengineering Centre (NIBEC), School of Engineering, Ulster University, Belfast, Northern Ireland UK; 2 School of Life Sciences, University of Warwick, Coventry, UK; 3 Institute for Global Food Security, School of Biological Sciences, Queen’s University Belfast, Belfast, UK; 4 Department of Biological and Biomedical Sciences, Glasgow Caledonian University, Glasgow, UK; 5 Dr B R Ambedkar Center for Biomedical Research, University of Delhi, Delhi, India; 6 Centre for Theoretical Physics and Natural Philosophy, Nakhonsawan Studiorum for Advanced Studies, Mahidol University, Nakhonsawan, Thailand; 7 Belfast School of Arts, Ulster University, Belfast, Northern Ireland UK

**Keywords:** Interdisciplinary studies, Policy and public health in microbiology, Antimicrobial resistance, Epidemiology, Environmental impact

## Abstract

Antimicrobial resistance (AMR) is a silent pandemic that presents a global challenge, urging researchers to develop innovative and transdisciplinary solutions. Our initiative aims to promote collaboration across science, engineering, economics, social sciences, and the arts to address the complex dimensions of AMR. We highlight the unique role of early-career researchers (ECRs) in advancing such cross-cutting approaches and conclude that empowering ECRs through equitable support and recognition is essential to sustaining innovation and mobilising communities against AMR.

## Abstract (web summary)

Antimicrobial resistance (AMR) is a global challenge. In this Perspective, authors highlight the unique role of early-career researchers (ECRs) in the transdisciplinary progress in AMR research.

## Introduction

Antimicrobial resistance (AMR) is a silent pandemic that threatens health systems, economies, and societies globally^1^. While AMR is fundamentally a medical challenge because it undermines treatment effectiveness, it is simultaneously driven by societal factors and therefore requires cross-disciplinary solutions^2^. Recent analyses have also highlighted a parallel crisis in the AMR research workforce, with declining numbers of professionals engaged in antimicrobial research and development (’brain drain’), driven by funding uncertainty and limited career progression opportunities^3,4^. This contraction of expertise indicates the urgency of sustaining and diversifying the talent pipeline, particularly through strengthened support for Early Career Researchers (ECRs) who represent the next generation of innovation and leadership in this field. Solutions must therefore extend beyond laboratory-based discovery to encompass social, behavioural, technological, and cultural dimensions. Addressing AMR requires more than collaboration within traditional scientific boundaries as it depends on bridging sectors from laboratory research and clinical practice to social science, arts, and policy engagement. Social science studies have demonstrated how prescribing practices, patient expectations, and community norms influence antibiotic use. Integrating these insights with biomedical and technological advances is essential for designing interventions that are context-sensitive, equitable, and sustainable^5,6^.

ECRs, here defined as researchers in the first independent stages of their careers, typically within eight years of PhD award or six years of first academic appointment^7^, or, in biomedical contexts, early stage investigators within ten years of terminal degree or clinical training and without a prior substantial independent award^8^, are not only contributing to this landscape, they are reshaping it. As the generation of scientists who will inherit the long-term consequences of AMR, ECRs are also uniquely positioned and personally invested in driving sustainable, transdisciplinary solutions. This perspective highlights how ECRs are redefining AMR research by bridging Science, Technology, Engineering, and Mathematics (STEM), social sciences and humanities, and proposes strategies to empower the next generation to sustain this momentum.

AMR is like a complex puzzle, with each piece representing a different aspect of society, culture, and human behaviour. To solve this puzzle, it is necessary to bridge together diverse perspectives and skills in a single cohort. Furthermore, the crux of the AMR crisis is the relationship between humans and microorganisms^9^. A diverse coalition of researchers, healthcare professionals, engineers, and policymakers are on the frontline creating new antibiotics, diagnostic tools, and innovative treatments. However, it is equally important to understand the complex behaviour of humans. For instance, how we use antibiotics, our attitudes towards healthcare, and the policies that govern medical practices are significant aspects that all contribute to the spread of resistance^10–12^.

This is where the humanities and social sciences come into play. Imagine a sociologist stepping into a rural community, uncovering the local beliefs and practices that influence antibiotic use. Or a philosopher questioning the ethical implications of distributing new drugs on who gets them first, and at what cost? These insights help shape public health campaigns that are not only scientifically sound but also culturally sensitive and ethically grounded. Evidence from Low and Middle Income Countries (LMIC) settings shows that cultural models of illness (e.g., viewing antibiotics as general ‘strong medicine’), trust in informal providers, household stockpiling, and pharmacy-level norms around non-prescription sales all influence self-medication with antibiotics^13^. Financial barriers to clinic access and perceptions that antibiotics accelerate recovery from everyday ailments further reinforce these practices. Interventions therefore need locally tailored combinations of community education, provider engagement, and regulatory action, alongside evaluation that captures social determinants and context^14,15^.

On the other hand, artists can use their craft to visualise microscopic worlds, provoke emotional engagement, and foster dialogue around AMR^16^. In recent times, arts-based engagement has increasingly been used to raise awareness of AMR and stimulate community discussion. For example, ART LAB, Cardiff University^17^curated commissioned artworks and a public competition culminating in an exhibition at Chapter Arts Centre, aiming to reach audiences beyond traditional STEM communities. Likewise, ‘Superbugs: A Pop-up Science Shop’ (Cardiff)^18,19^ transformed an empty retail unit into an immersive microbiology experience. Peer-reviewed accounts report substantial footfall during school holidays and qualitative evidence of learning and engagement. Museum late-night arts, microbiology events, have also demonstrated how integrated exhibitions can increase public understanding and participation among local communities^20^. Public-facing campaigns that pair creative engagement with clinician-facing stewardship are the ones most consistently associated with reduced antibiotic use at population level and single-channel awareness alone is rarely sufficient^21^. Some examples include Antibiotic Guardian (UK)^22^, whose evaluations show wide reach, improved knowledge, and self-reported behaviour change among participants over successive years. Taken together, creative formats ranging from exhibitions and pop-up science shops to theatre can broaden participation among audiences who might not otherwise engage with science.

**Fig. 1:**
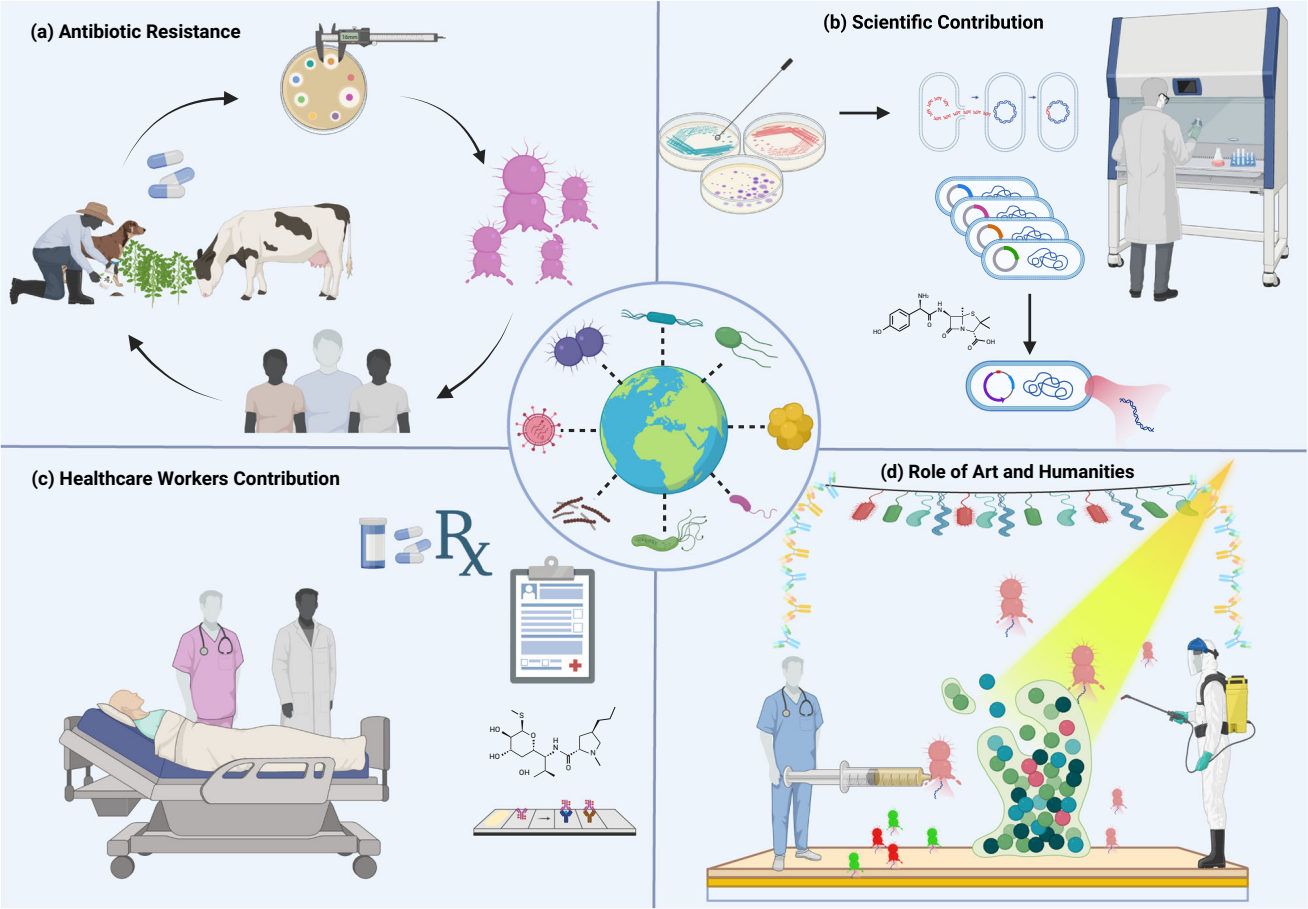
Global transdisciplinary perspective on AMR. **a** Illustration of the interconnected cycle of antibiotic resistance among humans, animals, and the environment, emphasizing the “One Health” concept. **b** Representation of laboratory research focused on understanding micro- bial mechanisms, drug discovery, and development of novel antimicrobial strategies. **c** Depiction of healthcare professionals implementing infection control measures, responsible antibiotic pre- scribing, and patient education to combat AMR. **d** Visualization of how art and humanities can raise awareness, promote public engagement, and encourage transdisciplinary understanding of antimicrobial resistance. The authors have applied the CC-BY 4.0 license to illustrations generated by Biorender: Created in BioRender. Chaudhary, V. (2026) https://BioRender.com/6c4a20f.

Integrating public-facing approaches with education and policy measures can help shift public expectations, alleviate prescribing pressures, and contribute to more sustainable antibiotic use. Recent work also highlights the value of structured patient and public involvement in shaping AMR research and policy, demonstrating that co-designed engagement initiatives can strengthen impact and legitimacy across social and clinical dimensions^23^. Future projects should include clear engagement metrics (e.g., reach, dwell time, pre/post knowledge, behavioural intentions) and, where feasible, link these to prescribing or dispensing trends to assess longer-term impact. Interaction between these fields, science, technology, social sciences, arts and humanities creates a holistic approach to AMR, as shown in Fig. 1 which highlight the transdisciplinary aspect of AMR.

Another aspect of AMR is that it is a prolonged battle requiring years of sustained effort, energy, and resources. In this context, the perspectives and ideas of ECRs are crucial. Therefore, we explore how ECRs can play significant roles in addressing the ongoing challenge of combating AMR. We propose an inclusive environment for ideas and initiatives, where ECRs can enhance existing technology, methods and knowledge (with complementary insights or collaborative efforts with established/senior investigators) to discover new scientific principles and invent advanced mitigation technologies and strategies to shape AMR research. Table 1 summarises the key enablers that can empower ECRs to address the multifaceted challenge of AMR. While ECRs often bring distinctive advantages such as flexibility to work across disciplines, openness to novel methodologies, and lived experience of an evolving research culture, these qualities complement, rather than replace, the expertise and institutional insight of established/senior principal investigators (PIs).

**Table 1:** Innovative aspects of early-career researchers in combating AMR

Unique Advantage	Areas/Actions	Impact/Examples
Agility and risk-taking	More open to exploring unconventional ideas and high-risk, high-reward projects, such as novel transdisciplinary approaches.	Rapidly testing innovative hypotheses or experimental methods that challenge traditional paradigms in AMR research.
Digital native proficiency	Greater familiarity with new digital tools, data analytics, and AI integration, enabling efficient analysis of complex datasets.	Developing smart biosensing platforms or using big data to predict microbial resistance patterns, thus accelerating diagnostic innovation.
Transdisciplinary collabora- tion	Eager to bridge fields, incorporating insights from technology, arts, humanities, and social sciences into their research.	Creating collaborative projects that merge scientific experiments with creative public outreach initiatives (e.g., art installations or interactive digital media campaigns).
Innovative communication	Willing to experiment with modern communication methods, including social media and digital storytelling, to trans- late scientific findings.	Engaging broader audiences through graphic novels, podcasts, or interactive webinars that demystify AMR and promote public understanding of the crisis.
Adaptive learning and flexibility	Quick to learn and adapt to emerging technologies and novel methodologies, often unencumbered by legacy systems or established routines.	Implementing agile research designs that integrate real-time monitoring tools or novel laboratory techniques to study microbial resistance in dynamic environments.

That aspects/ strengths are intended to complement and operate in collaboration with the expertise of senior and established researchers.

### Current progress

Tackling AMR requires foundational knowledge of microbe survival, resistance mechanisms, and environmental influences. ECRs are generating critical new insights through cutting edge discovery science^24^. They are applying tools like cryo-electron microscopy, proteomics, single cell sequencing, and artificial intelligence (AI) driven modelling to decode how microbes evade antimicrobial agents^25^. By revealing pathways critical for resistance and survival at the molecular and cellular level, they are identifying novel drug targets and informing the design of next generation therapies. Many ECRs are exploring alternative antimicrobial strategies, including bacteriophage therapy^26^, CRISPR based gene editing, and antimicrobial peptides derived from natural sources^27,28^. Others are ultilising machine learning to predict resistance patterns and guide treatment selection^29^. Importantly, they are situating this molecular research within broader ecological and evolutionary frameworks, investigating how environmental pressures, gene transfer, and agricultural practices influence resistance emergence. In redefining how we understand microbial behaviour, ECRs are laying the groundwork for targeted interventions^30^, more responsive public health strategies, and more effective AMR policies. Their work exemplifies how discovery science led by the next generation can directly impact global health.

**Fig. 2:**
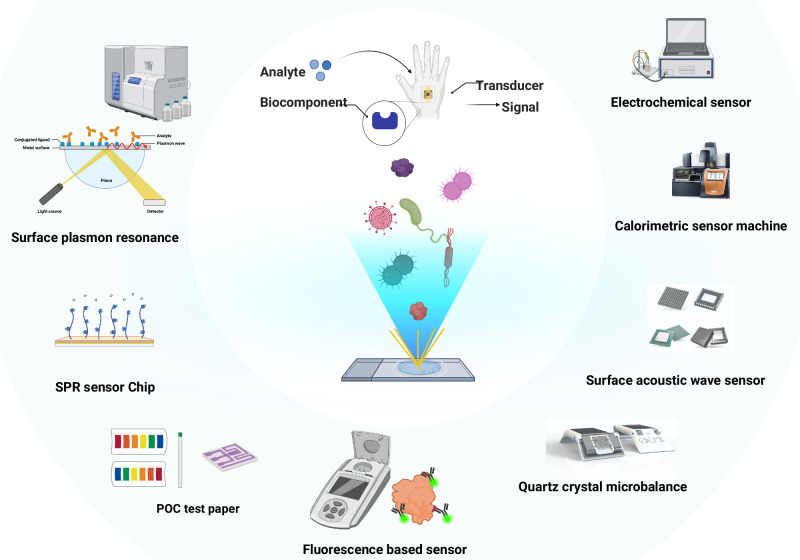
Overview of biosensing technologies for AMR research. This schematic illustrates various biosensor technologies used for detecting pathogens and biomolecules. It highlights key sensing mechanisms, including Surface Plasmon Resonance (SPR), electrochemical sensors, fluorescence-based sensors, calorimetric sensors, surface acoustic wave sensors, quartz crystal microbalance, and point-of-care (POC) test strips. The central concept demonstrates how an analyte interacts with a biocomponent, generating a measurable signal via a transducer. The authors have applied the CC-BY 4.0 license to illustrations generated by Biorender: Created in BioRender. Chaudhary, V. (2026) https://BioRender.com/7xi4zwz.

In the area of technology, new biosensing tools designed by ECRs are revolutionising the detection of bacteria and other pathogens, providing powerful methods to identify infectious agents with greater speed, accuracy, and sensitivity^31–33^. These tools employ various technologies such as plasmonics, fluorescence, electrochemical sensors, and microfluidics, enabling real-time detection and analysis of pathogens in complex samples, see Fig. 2. In the context of AMR, ECRs need to develop biosensors that have high sensitivity and selectivity, adaptable to both existing and pathogen of emerging concern. These sensors should also be portable, cost-effective, sustainable and disposable to prevent biological contamination. Moreover, biosensors must facilitate the study of microbial dynamics, including growth patterns, host interactions, and responses to environmental stressors. Real-time monitoring capabilities will be essential for enabling insights into microbial behaviour under various conditions, such as antibiotic exposure, to inform targeted treatments and uncover resistance mechanisms. Advanced technologies like surface plasmon resonance (SPR) and acoustic sensors recently developed by ECRs using vibrating solids exemplify progress in this field where the sound of bacteria hitting the surfaces is amplified^34^. Another critical application of these biosensors lies in high-throughput drug discovery^35,36^. By integrating with lab-on-a-chip devices or microplate readers, biosensors can rapidly screen thousands of compounds for antimicrobial activity. They can detect bacterial responses like growth inhibition or metabolic shifts, significantly accelerating the identification of effective drug candidates compared to traditional methods. However, the key scaling up of biosensing technology with high degree of versatility towards detection and analysis of microbes requires integration of AI. Essentially, AI will allow analyses of vast datasets generated by sensors, identify patterns, and predict microbial responses, enhancing diagnostic accuracy and speeding up drug development. In clinical settings, AI-driven biosensors may enable real-time monitoring and rapid decision-making, improving infection management and combating AMR more effectively^37^.

**Fig. 3:**
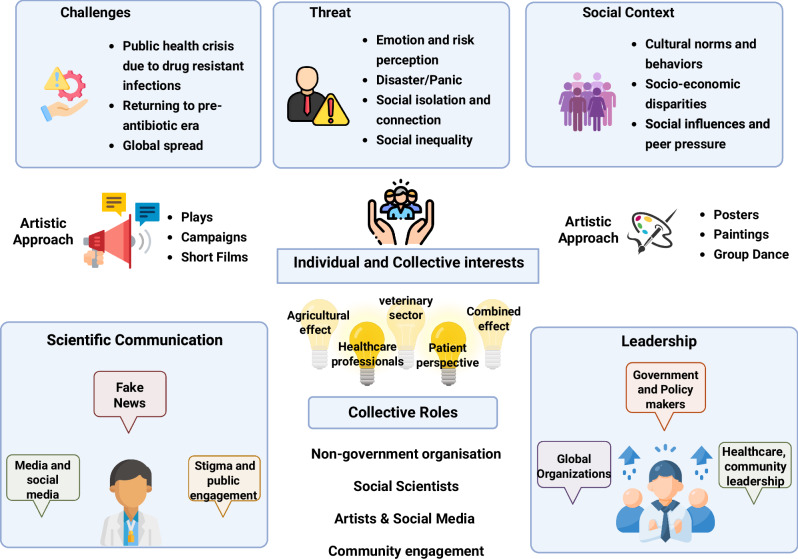
Framework of social sciences, humanities and arts in AMR. Conceptual framework illustrating how transdisciplinary collaboration and creative engagement address AMR. The central node represents shared individual and collective interests surrounded by six key domains: Challenges, Threat, Social Context, Scientific Communication, Leadership, and Artistic Approaches, that interact through communication, innovation, and community action. The central concept demonstrates how an analyte interacts with a biocomponent, generating a measurable signal via a transducer. The authors have applied the CC-BY 4.0 license to illustrations generated by Biorender: Created in BioRender. Chaudhary, V. (2026) https://BioRender.com/b9xxl5b.

The arts and humanities emerge as unexpected allies to enhance public understanding of AMR, Fig. 3. ECRs are increasingly embracing and championing the integration of the arts as a powerful tool to humanise AMR by transforming abstract data into compelling narratives that resonate with the public, fostering deeper understanding and more meaningful engagement with the AMR challenge^38^. For instance, a graphic novel could be developed where a hospital ward transforms into a battlefield, showing doctors and nurses battling a monstrous superbug. Similarly, a play that explores the anxieties of a family grappling with a drug-resistant infection could also assist in public awareness^39^. In organising such events, the role of visual arts is very important in assisting the understanding of AMR. The visual arts will aid the development of interactive art installations for museums that can become playful learning experiences for members of the community. For example, learning from the mandatory use of hand santiners during COVID-19 restrictions^40^ in schools, we can create a portrait of a giant, friendly monster-shaped handwashing station that encourages children to scrub away germs.

Indeed, some work in this aspect has already started to appear such as Sam Falconer’s illustration in Nature’s ‘Antimicrobial resistance: a silent pandemic’ collection, that uses dynamic, stylised infographics to demystify AMR mechanisms, making the invisible visible^41^. At Cardiff University, UK, the ART LAB blended arts and science and screened the documentary RESISTANCE, hosting expert panels, staging an AMR-themed exhibition,—to engage communities be yond traditional STEM^17^. Commissioned works by creative professionals like Drew Copus ab- stractly represented resistant microbes and transmission pathways, while public art entries fostered ownership and dialogue. These art-science collaborations bridge knowledge gaps, catalyse behavioural change, and democratise discourse on AMR. Furthermore, theatre productions and dance pieces can spark conversations about the overuse of antibiotics and the importance of responsible use and ignite community level behavioural changes that can make a real difference in combating AMR. For instance, Alex Howarth has made a powerful musical theatre production that dramatises the global crisis of AMR^42^. Briefly, their storyline intertwines two timelines: Alexander Fleming’s 1928 discovery of penicillin and a modern-day doctor, Jess, trying to save her partner from an AMR infection after routine surgery.

Ethnographic research, interviews, and quantitative studies can uncover the community level social behaviours and beliefs driving misuse and overuse of antibiotics. Social sciences critically explore how cultural norms, economic disparities, health inequalities and policy environments can help shape antibiotic use and contribute to AMR. Socioeconomic and behavioural factors such as out-of-pocket healthcare costs, poor governance, and weak regulatory frameworks contribute to antibiotic misuse and rising AMR^43^. This may further highlight the impact of public perceptions, trust in health institutions, interventions and socioeconomic factors on antibiotic practices. Insights from social sciences inform the development of targeted public health interventions and education programmes could further ensure that strategies to combat AMR are culturally sensitive and socially sustainable.

### ECRs as game changers in the AMR fight

AMR is a future-facing challenge, and ECRs are the generation that will inherit its consequences. In this context, ECRs are uniquely poised to drive innovation against AMR, utilising their agility and transdisciplinary fluidity to overcome traditional barriers. Unencumbered by entrenched paradigms, they have pioneered CRISPR-based rapid diagnostics^44^ and AI-driven antibiotic discovery, famously identifying “halicin” via deep learning^45^. Their mastery of big-data platforms enables real-time fusion of genomic, clinical, environmental, and socio-behavioural datasets, transforming static surveillance into predictive models that preempt resistance hotspots. By championing FAIR (Findable, Accessible, Interoperable, Reusable) data principles^46^, ECRs dismantle silos between microbiology, engineering, public health, and the social sciences, catalysing truly transdisciplinary consortia.

However, ECRs face formidable hurdles, key challenges are shared in Table 2. Traditional grant mechanisms seldom fund translational work beyond proof-of-concept, leaving a “valley of death” at the academia–industry interface. Regulatory pathways for novel diagnostics are opaque and protracted, demanding expertise in regulatory science that many ECRs have yet to develop. In low- and middle-income countries (LMICs), resource constraints, cultural and linguistic barriers, and community mistrust can stall field deployments, requiring time-intensive co-design with local stakeholders^47^. Moreover, proprietary data platforms and inconsistent interoperability standards fragment critical datasets, undermining the holistic analyses ECRs strive to deliver^48^. Despite these challenges, ECRs harness entrepreneurial fellowships, open-source toolkits, and global peer-mentoring networks to persevere bridging from bench to field, and from data to policy, they are redefining AMR stewardship for the coming decades.

**Table 2:** Critical challenges to transdisciplinary AMR action for ECRs

Challenge	Perspective
Navigating regulatory pathways	Approval processes for novel diagnostics (e.g., CRISPR-based sensors) vary widely between regions, lack clear guidance, and can take years requiring ECRs to build regulatory expertise or partner with experienced mentors early.
Securing translational funding	Standard academic grants seldom support scale-up beyond proof-of-concept, while industry may view AMR innovation as high- risk. ECRs must diversify funding, combining small pilot awards, translational fel- lowships, and accelerator programmes to bridge this “*valley of death*.”
Engaging communities in LMICS	Resource constraints, language barriers, and mistrust of outsiders can stall interventions. ECRs should allocate time for co-design with local stakeholders, employ culturally relevant outreach, and seek institutional support for fieldwork logistics.
Intgerating disparate data streams	Proprietary platforms and inconsistent standards mean laboratory, surveillance, and socio-behavioural data often live in silos. ECRs can overcome this by adopting open-source tools, championing Findable, Accessible, Interoperable, and Reusable **(**FAIR) principles, and advocating for interoperable data-sharing policies.

### Empowering the next generation to tackle AMR

To fully harness the potential of ECRs in the fight against AMR, we must build structures that empower rather than constrain. It requires academic and non-academic environments that value curiosity-driven, cross-sectoral, and collaborative work. Training should be redesigned to equip ECRs with more than technical skills. We must foster systems thinking, public engagement, and ethical reflexivity. Opportunities for early exposure to transdisciplinary teamwork, stakeholder engagement, and real-world policy challenges are essential. Formal mentoring and peer networks must also be strengthened to combat isolation and encourage bold experimentation, key priorities of the Futures AMR Network (FAN- https://www.futuresamr.co.uk)^49^ and other similar/sister networks. Global inequities in research opportunity must also be addressed. This includes tackling visa and mobility barriers, creating equitable partnerships with LMICs, and supporting inclusive career pathways for researchers from underrepresented backgrounds. Similar disparities have been observed in other research domains, where early-career scholars face structural inequalities linked to geography, resources, and recognition, - patterns that mirror those in AMR research and highlight the need for globally equitable support mechanisms^50^. Empowering ECRs means trusting them with leadership, decision-making roles, and space to fail and learn. It means amplifying their voices in AMR policy, funding, and strategy development. Because when ECRs thrive, the AMR field as a whole becomes more dynamic, inclusive, and better equipped to tackle future threats.

Supporting the next generation of researchers is vital for sustaining transdisciplinary progress in AMR research. As we have discussed, ECRs are often the driving force behind innovative, cross-sector collaborations that bridge the boundaries of science, technology, social sciences, and the arts. Their capacity to adapt, experiment with new methodologies, and connect across disciplines places them in a unique position to advance creative and integrative approaches to complex health challenges such as antimicrobial resistance. However, despite this potential, many ECRs face persistent structural barriers including limited recognition, short-term contracts, unstable funding, restricted access to leadership opportunities, and unequal participation in decision-making processes.

Several funding bodies have recognised the importance of supporting transdisciplinary training and career development for researchers tackling AMR. In the UK, UK Research and Innovation (UKRI) has invested in transdisciplinary AMR networks and capacity-building initiatives that integrate biomedical, environmental, social, and creative disciplines. The Medical Research Foundation’s National PhD Training Programme in AMR Research^24^ provides a model for structured doctoral support, fostering collaboration across institutions and sectors. Internationally, Wellcome has established and expanded programmes aimed at strengthening global AMR research capacity and building a sustainable talent pipeline through long-term, cross-institutional partnerships. Collectively, these initiatives illustrate the growing recognition that addressing AMR requires not only scientific innovation but also investment in people and transdisciplinary training pathways. Given that AMR is a multi-generational challenge and represents a continuous threat that will shape the lives of generations to come. Nevertheless, challenges remain in ensuring continuity of support and equitable access to resources for emerging researchers across regions and disciplines.

To fully harness their potential, there is a pressing need for stronger institutional and policy-level support. Dedicated ECR-focused funding streams, transdisciplinary fellowships, and mentorship networks that connect emerging and senior researchers across sectors can provide the stability and confidence needed to pursue ambitious, long-term research agendas. Embedding mentorship as a core element of research capacity strengthening, particularly when grounded in reciprocal, value-driven practices, has been shown to enhance career development and equity in global health research^51^. Recognising ECRs not only as contributors but as co-leaders and knowledge brokers in AMR research who are capable of linking laboratory science with social, cultural, and policy insights will encourge a more collaborative and sustainable research ecosystem. Emerging models of collaborative training and mentorship demonstrate how shared responsibility between institutions, funders, and senior researchers can strengthen ECR development. For example, the Wellcome-funded CAMO-Net (Community AMR Network)^52^ provides an interdisciplinary platform for capacity building, connecting researchers across regions to exchange knowledge, co-design community engagement projects, and foster equitable global partnerships. Similarly, the Fleming Initiative Fellowships^53^, including those focusing on artificial intelligence and innovation, aim to cultivate leadership skills and translational expertise among early-career scientists and clinicians. These frameworks exemplify how coordinated investment and mentorship can empower ECRs to operate effectively within the complex, multi-sector environment required to address AMR.

Furthermore, promoting inclusive authorship practices and fair attribution in multi-author and cross-institutional projects is essential to ensure that ECR contributions are visible and valued. Embedding ECR representation in strategic planning, funding review panels, and AMR policy advisory groups can help democratise research leadership and empower the next generation to shape the global AMR agenda. By investing in the capacity, recognition, and leadership of ECRs, the AMR research community can build a more dynamic, equitable, and future-ready system of knowledge creation that bridges disciplines and drives lasting impact. The reflections and recommendations presented here extend beyond AMR. While AMR provides a compelling case study of the need for cross-sector collaboration, many of the lessons are generalisable to ECRs development more broadly. Structured mentorship, equitable recognition, and stable funding are universal enablers of research capacity, while interdisciplinary and creative approaches can invigorate innovation across domains. Promoting inclusive leadership and embedding ECR voices in decision-making processes, both within institutions and funding bodies, can enhance engagement, retention, and impact across the global research ecosystem. These considerations are especially critical in the AMR field, where retaining ECRs requires sustained investment in environments that make long term engagement both viable and attractive, especially as AMR stands as a global health challenge of profound and continuing societal importance.

### Conclusion and future recommendations

Tackling AMR requires more than scientific discovery and essentially demands a sustained cultural and institutional shift towards interdisciplinary collaboration and shared responsibility. This perspective has highlighted how ECRs are contributing to and redefining this shift, integrating approaches from STEM with those from the social sciences, arts, and humanities. Their work reflects a broader change in research culture: one that values creativity, inclusivity, and collaboration across sectors. However, sustaining this progress requires more than individual motivation and we suggest for coordinated action at multiple levels. For instance, funders can play a transformative role by creating long-term, flexible funding schemes that encourage interdisciplinary projects and provide secure career pathways for emerging researchers. Universities and research institutions should embed mentorship, equitable recognition, and cross-sector training as core components of research culture, ensuring that ECRs are supported not only as contributors but as co-leaders of innovation. Policymakers and international organisations should actively involve ECRs in AMR strategy design, global networks, and advisory boards, ensuring that diverse perspectives inform policy and implementation. As policymakers, governments, and international bodies have already positioned AMR as a global societal health crisis and a silent pandemic, this recognition must now be matched and must translate into a serious and sustained commitment to strengthening the financial incentives that support long term engagement in AMR research for current and future ECRs.

As emphasised by the European University Association, sustained investment in early-career researchers is not only vital for disciplinary renewal but also for the long-term vitality of higher education and global research ecosystems^54^. By recognising and resourcing ECR leadership today, we not only accelerate progress on AMR but also help shape a more resilient, equitable, and connected research ecosystem. Investing in the next generation of AMR researchers is an investment in the sustainability of global health and the future of collaborative science. Building on this shared responsibility, across career stages, disciplines, and institutions, will be essential to generate the creativity and collective momentum needed to confront the AMR crisis.
